# Balancing Stone Prevention and Kidney Function: A Therapeutic Dilemma

**DOI:** 10.3390/jcm14113678

**Published:** 2025-05-23

**Authors:** Natalia Stepanova

**Affiliations:** 1 State Institution “O.O. Shalimov National Scientific Center of Surgery and Transplantology of the National Academy of Medical Science of Ukraine”, 03680 Kyiv, Ukraine; n.stepanova@nephrocenter.com; 2 Dialysis Medical Center LLC “Nephrocenter”, 03057 Kyiv, Ukraine

**Keywords:** nephrolithiasis, chronic kidney disease, urinary calculi, kidney function, prevention, precision medicine

## Abstract

Managing nephrolithiasis in chronic kidney disease (CKD) poses a therapeutic challenge: preventing stone recurrence while preserving kidney function. Standard urological interventions and preventive strategies, such as high fluid intake, thiazides, and potassium citrate, cut recurrence by 50–60% in healthy kidneys but risk fluid overload, hyperkalemia, and diminished efficacy in CKD as glomerular filtration rate (GFR) declines. Often, stone prevention and CKD care are addressed separately, leaving clinicians without unified guidance for this rising patient group. This review explores the bidirectional relationship between nephrolithiasis and CKD, integrating pathophysiology and therapeutic strategies into a practical, decision-oriented framework. It offers tailored interventions based on GFR category, stone type, and comorbid conditions, emphasizing the potential for dual-purpose therapies. Going beyond previous reviews, it connects clinical practice with existing research gaps, offering tools to balance outcomes and guide future studies.

## 1. Introduction

Nephrolithiasis affects approximately 10–12% of the global population, with recurrence rates exceeding 50% within ten years of the initial event [1,2]. Its prevalence and incidence have increased markedly in recent decades, reaching up to 13% in Europe [3], 15% in North America, and 20% in some regions such as Saudi Arabia [4,5]. Between 2000 and 2019, global kidney stone-related incident cases, deaths, and disability-adjusted life years rose by 26.7%, 60.3%, and 34.5%, respectively, underscoring the growing public health burden of this condition [6].

Concurrently, chronic kidney disease (CKD) impacts over 13% of adults worldwide, representing a significant and growing health challenge [7,8]. Currently impacting over 700 million individuals worldwide, CKD has emerged as one of the fastest-rising causes of premature mortality [8]. The increasing prevalence of nephrolithiasis and CKD mirrors the global rise in metabolic disorders such as obesity, diabetes, and hypertension, all of which are strongly associated with both conditions [8,9,10]. Recent evidence from a population-based analysis of NHANES 2007–2020 further supports this link. Individuals in the highest stages of cardiovascular–kidney–metabolic syndrome (defined by an eGFR < 60 mL/min/1.73 m^2^) were significantly more likely to report a history of nephrolithiasis compared to those in earlier stages, highlighting the shared metabolic and inflammatory pathways underlying both conditions [11].

Once considered a localized urological disorder, nephrolithiasis is now understood to be a systemic condition associated with metabolic disturbances, chronic inflammation, and tubular injury, which are key drivers of CKD [12]. Mounting evidence supports a strong association between nephrolithiasis and progressive kidney function decline. A seminal study reported a 50–67% increased likelihood of clinically diagnosed CKD among stone formers, independent of confounding factors [10]. Similarly, recent cohort analyses further confirm this association, documenting hazard ratios (HRs) for CKD development ranging from 1.29 to 1.82 in stone formers compared to controls [13,14,15]. These associations are thought to arise from both direct and indirect mechanisms. Recurrent stones, obstruction, and infection can cause sustained renal injury, while underlying metabolic abnormalities may further accelerate CKD progression [2]. Conversely, CKD itself can promote stone formation through alterations in urinary composition, acid–base balance, and solute excretion [16], creating a complex interplay that poses a therapeutic challenge for clinicians.

Despite the growing recognition of this interplay, epidemiological data quantifying the proportion of end-stage kidney disease (ESKD) cases directly attributable to nephrolithiasis remain limited [4,10,15]. This gap in evidence underscores a broader issue in clinical practice: while urologic care has traditionally focused on stone prevention and clearance, preservation of kidney function often receives less attention. This imbalance presents a therapeutic dilemma, particularly in patients with coexisting CKD. Preventive strategies such as increased fluid intake, dietary modification, and pharmacologic interventions, including thiazide diuretics and potassium citrate, are well-established in stone management. However, their use in patients with impaired kidney function raises important concerns regarding safety, efficacy, and long-term renal outcomes. This tension is further complicated by the fact that kidney function monitoring is not always fully integrated into routine urologic care, especially when the focus remains primarily on stone reduction. Consequently, patients might receive interventions that, although effective in reducing stone burden, have the potential to negatively impact renal outcomes if not appropriately tailored.

To address this knowledge gap, this review synthesizes current evidence at the intersection of nephrolithiasis and CKD, where a lack of consensus on tailoring interventions across the spectrum of kidney function complicates clinical management [17,18]. Details of the literature search and study selection are provided in Supplementary File S1. Unlike prior reports that separately address stone recurrence in healthy kidneys or CKD management, this review examines the dual impact of therapeutic strategies on stone risk and renal outcomes. It analyzes the mechanisms, benefits, and potential harms of widely used interventions to clarify this complex balance. Furthermore, it structures evidence from current and emerging strategies, highlighting inconsistencies in clinical translation and the need for personalized approaches to optimize renal outcomes for patients with nephrolithiasis and CKD. Addressing this therapeutic challenge is both a clinical imperative and an opportunity to advance integrated care for this population.

## 2. Pathophysiology of the Nephrolithiasis–CKD Cycle

**Figure 1:**
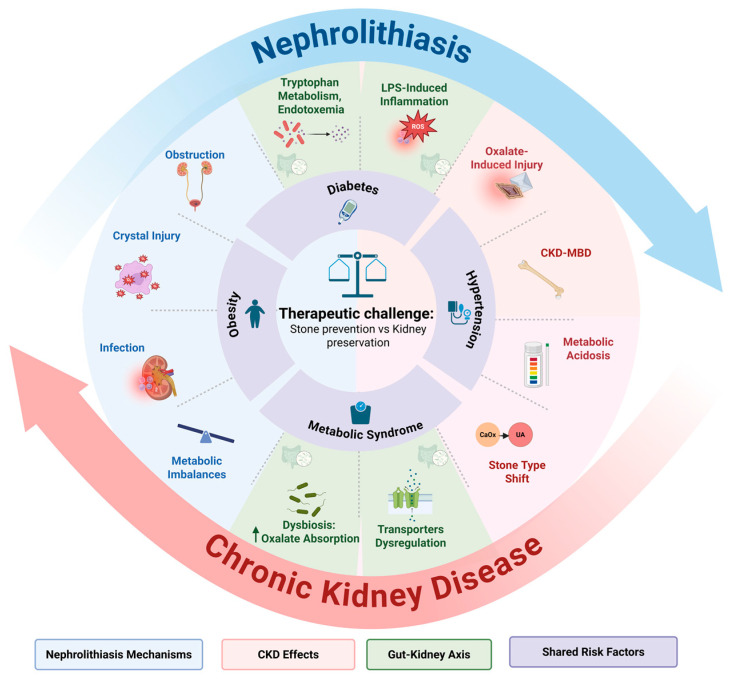
Pathophysiology of the nephrolithiasis–CKD cycle. The diagram illustrates the bidirectional relationship between nephrolithiasis and CKD. Nephrolithiasis drives kidney damage through mechanisms such as obstruction, crystal injury, infection, and metabolic and chemical imbalance. CKD alters stone formation via oxalate-induced injury, metabolic acidosis, and stone-type shifts. The gut–kidney axis amplifies both processes through dysbiosis (oxalate absorption by gut bacteria), tryptophan metabolism, and endotoxemia (LPS, indoxyl sulfate production, decreased citrate), contributing to inflammation and fibrosis. Shared risk factors contribute to both conditions, leading to the therapeutic challenge of balancing stone prevention with kidney function preservation. Abbreviations: CKD-MBD, chronic kidney disease mineral bone disorders; LPS, lipopolysaccharide.

The challenge of balancing kidney stone prevention with kidney function preservation arises from the complex, bidirectional relationship between nephrolithiasis and CKD. Nephrolithiasis drives kidney damage, accelerating CKD progression, while CKD alters stone formation in both predictable and paradoxically inverse ways, thus complicating the risk profile. Although this dynamic has been partially documented in prior studies [12,16], this section summarizes the shared risk factors, mechanisms of stone-induced kidney injury, the dual role of CKD in stone formation, and the systemic influence of the gut–kidney axis, providing a foundation for understanding this therapeutic dilemma (Figure 1).

### 2.1. Shared Risk Factors

Nephrolithiasis and CKD converge through shared risk factors such as hypertension, diabetes mellitus, obesity, and metabolic syndrome, which synergistically elevate the incidence and severity of both conditions via overlapping pathophysiological mechanisms.

#### 2.1.1. Hypertension

Hypertension is associated with a 1.5- to 2-fold increased risk of nephrolithiasis [9,19] and represents a major contributor to CKD [20,21]. Pathophysiologically, hypertension activates the renin–angiotensin–aldosterone system (RAAS), leading to increased angiotensin II levels, which enhance proximal tubular sodium reabsorption while reducing calcium excretion, ultimately predisposing to hypercalciuria [21,22]. Additionally, reduced renal perfusion and microvascular injury concentrate urinary solutes, facilitating calcium stone formation. In the context of CKD, hypertension induces glomerular hyperfiltration and shear stress, promoting endothelial dysfunction, inflammation, and tubulointerstitial fibrosis through the transforming growth factor-beta (TGF-β) signaling pathway [23]. These maladaptive changes impair tubular function, further increasing nephrolithiasis risk and perpetuating a pathological cycle.

#### 2.1.2. Diabetes Mellitus

Type 2 diabetes mellitus elevates nephrolithiasis risk by 1.3–1.7 times [24] and accounts for up to 40% of CKD cases [25]. Insulin resistance impairs ammoniagenesis by downregulating ammonium production in proximal tubules, lowering urinary pH, and favoring uric acid crystallization [26]. Hyperglycemia-induced glycosuria can increase urinary calcium excretion through altered renal tubular handling, potentially contributing to calcium oxalate (CaOx) stone formation, though the specific role of the sodium–calcium exchanger (NCX1) in this process remains unclear and requires further investigation [24]. In CKD, diabetes drives glomerular hyperfiltration, mesangial expansion, and podocyte loss through advanced glycation end-products (AGEs) and their activation of the receptor for AGEs, culminating in diabetic nephropathy [25]. Oxidative stress amplifies these pathological mechanisms by promoting inflammation, fibrosis, and endothelial dysfunction, accelerating both nephrolithiasis and CKD progression [23,25,27].

#### 2.1.3. Obesity

Obesity (BMI ≥ 30 kg/m^2^) has been shown to significantly contribute to both nephrolithiasis and CKD [28,29]. It increases 1.25-dihydroxyvitamin D synthesis via adipose tissue, elevating intestinal calcium absorption and causing hypercalciuria [30]. Insulin resistance reduces urinary pH, while visceral fat enhances oxalate and uric acid production via purine metabolism, promoting stone formation [30,31]. In CKD, obesity induces glomerular hyperfiltration, activating the mTOR pathway and glomerulosclerosis. Adipokine dysregulation, such as elevated leptin and reduced adiponectin, amplifies inflammation, while lipotoxicity from free fatty acids drives mitochondrial dysfunction and fibrosis [29,32]. These effects compound with hypertension and diabetes, worsening renal outcomes.

#### 2.1.4. Metabolic Syndrome

Metabolic syndrome (MetS) significantly increases nephrolithiasis and CKD risk by 1.3–2-fold, depending on the presentation of its components [33,34]. Insulin resistance and intracellular acidosis reduce citrate synthase activity, causing hypocitraturia, while low urinary pH and elevated uric acid from dyslipidemia promote uric acid stones [35]. Additionally, MetS-related insulin resistance and obesity can elevate urinary oxalate excretion by enhancing endogenous oxalate synthesis and intestinal absorption, particularly in the presence of dietary oxalate or gut dysbiosis [36]. High oxalate levels promote calcium oxalate stone formation and directly injure renal tubular cells, triggering inflammation and fibrosis. In CKD, MetS accelerates progression through endothelial dysfunction, chronic inflammation, and oxidative stress, activating TGF-β/Smad and pro-fibrotic pathways [30,32,33]. Oxalate further exacerbates CKD by depositing in renal tissue, amplifying oxidative stress and interstitial damage [37]. These factors also elevate albuminuria and cardiovascular risk, with stone formers facing a 20–40% higher incidence of cardiovascular events [14,22].

### 2.2. Mechanisms Linking Nephrolithiasis to Kidney Damage

Nephrolithiasis can precipitate kidney damage through mechanical, crystalline, infectious, metabolic mechanisms, and gut–kidney interactions, each contributing to the progression of kidney injury and elevating CKD risk. Importantly, nephrolithiasis and CKD interact dynamically, with kidney stones often triggering acute-on-chronic kidney injury [10,12]. Acute kidney injury (AKI)-on-CKD arises when acute stone-related events-such as urinary tract obstruction, crystal-induced tubular injury, or infection, occur in the context of already reduced renal functional reserves [10,38]. These acute episodes can lead to persistent tubular dysfunction, unresolved inflammation, and progressive interstitial fibrosis, creating a cycle of ongoing nephron loss and further CKD progression [12,38]. AKI-on-CKD thus represents a critical inflection point in CKD’s natural history, shifting a stable chronic condition toward rapid progression and ESKD [39]. The specific stone-related injury mechanisms are described below.

#### 2.2.1. Obstruction

Ureteral stones cause obstructive nephropathy, a primary driver of nephrolithiasis-associated AKI, accounting for 1–2% of adult AKI cases [38,40]. This obstruction elevates intratubular pressure, triggering renal vasoconstriction within 6–12 h via angiotensin II type 1 (AT1) receptor activation and tubuloglomerular feedback, reducing GFR by up to 50% in unilateral ureteral obstruction models [40,41]. Molecularly, angiotensin II activates the AT1 receptor, upregulating NADPH oxidase to produce reactive oxygen species (ROS), which impair endothelial nitric oxide synthase (eNOS) activity, exacerbating ischemia [40,41]. Prolonged obstruction (days to weeks) shifts this acute response to chronic injury: ROS and hypoxia induce tubular epithelial cell apoptosis via caspase-3 activation, while interleukin-1β (IL-1β) and tumor necrosis factor-alpha (TNF-α) from infiltrating macrophages upregulate TGF-β/Smad signaling, leading to interstitial fibrosis and tubular atrophy [40,41,42]. Clinically, recurrent obstruction correlates with a 1.5–2-fold increased CKD risk [43], as impaired solute clearance concentrates stone-forming ions (e.g., Ca^2+^, C_2_O_4_^2−^), perpetuating stone formation.

#### 2.2.2. Crystal-Induced Tubular Injury

The crystal components of kidney stones exert direct toxic effects on renal tubular cells, leading to oxidative stress, inflammation, and fibrosis, ultimately contributing to CKD [38,40,44]. Supersaturation of urine with stone-forming substances, such as oxalate and uric acid, drives crystal nucleation [45,46]. Calcium oxalate monohydrate (COM) crystals have been shown to adhere to renal tubular epithelial cells, compromising the epithelial barrier and initiating cellular injury [47]. This adhesion, facilitated by surface interactions with cellular receptors, promotes crystal retention and subsequent damage to the tubular lining [48]. Large crystal aggregates obstruct tubular flow, causing mechanical injury, while smaller crystals are endocytosed, leading to mitochondrial dysfunction and lysosomal rupture [49,50].

Mitochondrial impairment increases ROS production via NADPH oxidase and xanthine oxidase pathways, overwhelming antioxidant defenses and causing oxidative stress [51,52]. This leads to lipid peroxidation, protein carbonylation, and DNA damage. ROS also activate apoptotic pathways via Bax/Bcl-2 imbalance and caspase-9 activation [52,53]. Additionally, NLRP3 inflammasome activation, triggered by ROS-mediated potassium efflux and lysosomal rupture, leads to IL-1β and IL-18 release, exacerbating inflammation and tubular injury [54]. Persistent inflammation promotes fibrosis through TGF-β/Smad2/3 signaling, leading to fibroblast activation and extracellular matrix deposition [52,53,54]. Consequently, crystal-induced injury creates a self-perpetuating cycle, where damaged tubular cells release debris that facilitates further crystal aggregation, worsening epithelial damage and fibrosis and reinforcing kidney stone formation.

#### 2.2.3. Infection

Nephrolithiasis-associated infections, particularly with struvite (magnesium ammonium phosphate) stones, significantly contribute to kidney damage by acting as niduses for bacterial colonization, most commonly by urease-producing pathogens such as *Proteus mirabilis*, *Escherichia coli*, and *Klebsiella pneumoniae* [55,56]. These bacteria hydrolyze urea into ammonia and carbon dioxide via urease, alkalinizing urine (pH > 7.2) and promoting struvite crystal precipitation, which exacerbates stone growth and obstructs urinary flow [56]. Beyond urease producers, any urinary tract infection (UTI), such as those involving *Staphylococcus aureus* or *Pseudomonas aeruginosa*, can exacerbate kidney injury, as shown in CKD cohort studies [57,58]. The resulting infection triggers acute pyelonephritis, characterized by neutrophil infiltration and release of pro-inflammatory cytokines such as IL-1β, IL-6, and TNF-α via Toll-like receptor 4 (TLR4) activation on tubular epithelial cells [59,60]. Bacterial lipopolysaccharides (LPS) bind TLR4, upregulating nuclear factor-kappa B (NF-κB) signaling, which amplifies cytokine production and recruits macrophages via monocyte chemoattractant protein-1 (MCP-1) [59,61]. This inflammatory cascade activates matrix metalloproteinases (MMPs), such as MMP-9, which degrade tubular basement membranes and promote epithelial-to-mesenchymal transition (EMT), a precursor to interstitial fibrosis [41,62].

#### 2.2.4. Metabolic and Chemical Imbalance

Nephrolithiasis is associated with metabolic and chemical imbalances that both precipitate stone formation and exacerbate kidney damage in a bidirectional cycle [38,63,64]. The imbalances, such as hyperoxaluria, hypocitraturia, hypercalciuria, hyperuricosuria, and acid–base dysregulation, alter the urinary composition, promoting crystal nucleation, while simultaneously inducing tubular toxicity, oxidative stress, and inflammation that impair kidney function [63,64,65].

- Hyperoxaluria (>44 mg/day), from dietary intake, enteric malabsorption, or primary hyperoxaluria, increases urinary supersaturation, fostering COM crystal formation [66,67]. Oxalates trigger NADPH oxidase-mediated ROS production, which disrupts mitochondrial membrane potential and activates caspase-9-mediated apoptosis [65,66,67]. Elevated oxalate also upregulates angiotensin II via AT1 receptors, enhancing ROS and promoting tubular injury and interstitial fibrosis through TGF-β/Smad3 signaling [37,54,67]. Clinically, hyperoxaluria correlates with a 33% higher CKD progression risk and 45% higher end-category kidney disease (ESKD) risk [68], as impaired oxalate clearance concentrates it further.
- Hypocitraturia (<320 mg/day), often secondary to metabolic acidosis (HCO_3−_ < 22 mmol/L) or CKD-induced proximal tubular dysfunction, reduces citrate’s ability to chelate calcium and inhibit crystal growth [63,69]. Acidosis upregulates the Na^+^/dicarboxylate cotransporter (NaDC-1), lowering urinary citrate and increasing ROS, promoting COM and uric acid stones with IL-6/TNF-α-driven fibrosis [63,70]. In CKD, citrate depletion accelerates, creating a feedback loop where reduced GFR worsens hypocitraturia [69].
- Hypercalciuria (defined as >250 mg/day in women and >300 mg/day in men), whether idiopathic or secondary, is a significant risk factor for calcium oxalate and calcium phosphate stone formation [71,72]. Excess urinary calcium can bind to tubular epithelial cells via integrin receptors, triggering oxidative stress and activating the NLRP3 inflammasome, which leads to the release of IL-1β and amplifies inflammation [71,72,73]. Chronic hypercalciuria also contributes to the formation of Randall’s plaques, which are interstitial calcium phosphate deposits in the renal papillae [72,73]. These plaques can erode into the collecting system, serving as a nidus for stone formation. Over time, this process can cause tubulointerstitial damage and is associated with an increased risk of CKD [74].
- Hyperuricosuria (>700 mg/day), often from purine-rich diets or gout, typically acidifies urine (pH < 5.5), promoting uric acid crystal precipitation [75,76]. These crystals can obstruct tubules and activate NLRP3 inflammasome via xanthine oxidase-driven ROS [75,76]. However, in specific contexts, elevated uric acid levels may be associated with better renal outcomes [77], possibly due to its extracellular antioxidant properties, scavenging ROS, and potentially reducing oxidative stress-mediated tubular injury [78]. This protective effect is most evident in early-category CKD patients with preserved GFR, though the exact magnitude of risk reduction remains unclear. This protective effect diminishes in advanced CKD, where reduced clearance concentrates uric acid, shifting stone composition toward uric acid and amplifying damage via NLRP3 and MCP-1-driven fibrosis, reflecting a bidirectional dynamic [75,76].

#### 2.2.5. Gut–Kidney Axis Interactions

The gut–kidney axis amplifies nephrolithiasis-induced kidney damage through microbiota dysregulation and altered solute transport, distinct from mechanical and metabolic mechanisms [79,80]. Gut microbiota dysbiosis, prevalent in metabolic disorders, enhances intestinal oxalate absorption via modified *SLC26A1* and *SLC26A6* activity, driving calcium oxalate crystallization and tubular oxidative stress through NADPH oxidase pathways [79,81]. Reduced populations of short-chain fatty acid (SCFA)-producing bacteria, such as *Lactobacillus* and *Akkermansia*, diminish anti-inflammatory and antifibrotic effects [80,82,83], while uremic toxins like indoxyl sulfate, derived from microbial tryptophan metabolism, intensify oxidative stress and activate TGF-β/Smad3 signaling [84,85,86]. Endotoxemia from gut-derived lipopolysaccharides triggers TLR4/NF-κB signaling, elevating pro-inflammatory cytokines that promote crystal adhesion and interstitial fibrosis [81,82]. In CKD, declining GFR impairs *SLC26A1*-mediated oxalate secretion, increasing urinary oxalate, while acidosis disrupts *SLC13A2/SLC13A3* (NaDC-1/NaDC-3), reducing citrate levels and favoring lithogenesis [72,87]. These gut–kidney interactions exacerbate kidney injury beyond local stone effects, highlighting a systemic dimension to the nephrolithiasis–CKD interplay that complicates therapeutic balancing.

### 2.3. Dual Role of CKD in Kidney Stone Formation

CKD influences nephrolithiasis through biochemical and metabolic changes that both increase overall stone risk and alter stone-type prevalence, creating unique challenges for prevention [12,16].

As CKD progresses, it alters urinary biochemistry in ways that both promote and inhibit stone formation. In early CKD (G1, G2), calcium oxalate stones predominate (69.3%), followed by calcium phosphate stones (11.9–19.7%) [88]. However, as kidney function declines in advanced CKD (G3-G5), there is a shift in stone composition, with uric acid stones increasing significantly (28.7%) while calcium oxalate stones decrease [88].

Metabolic acidosis, a common feature of CKD (serum bicarbonate < 22 mmol/L), disrupts renal tubular function, promoting stone formation and tubular damage through proximal and distal effects [69,89]. In the proximal tubule, acidosis upregulates NaDC-1, increasing citrate reabsorption and reducing urinary citrate [90,91]. Concurrently, xanthine oxidase activity elevates ROS and uric acid production, intensifying oxidative stress and inflammation. Distally, acidosis can cause distal renal tubular acidosis, impairing H^+^ excretion via defective H^+^-ATPase or H^+^/K^+^-ATPase, preventing urine acidification below pH 5.3 and driving calcium phosphate stone nucleation [90,91]. These alterations are associated with a higher risk of CKD progression [92,93]. Tubular injury concentrates stone-forming solutes like Ca^2+^ and uric acid, while CKD exacerbates acidosis, perpetuating a harmful cycle.

Urinary biochemical changes across GFR categories play a crucial role in shifting stone risk and composition [16,88]. In early CKD, urine pH is generally normal to slightly acidic. However, urinary calcium excretion begins to decrease even in the early categories, correlating with declining GFR [94]. As CKD advances, metabolic acidosis leads to more acidic urine (pH < 5.5), decreased urinary calcium excretion, and significantly reduced citrate levels (30–40% decrease) [16,87,88]. These changes, along with decreased magnesium and phosphorus excretion, alter the balance of stone-promoting and stone-inhibiting factors [16,88]. Acidity and low calcium saturation offer a paradoxical protective effect against calcium-based stones, thus reducing their incidence in advanced CKD. However, the risk of uric acid stones increases due to persistently acidic urine despite lower uric acid excretion [16] (Table 1).

Other central mechanisms underlying CKD-associated changes in stone risk include reduced stone precursor excretion and mineral and bone disorders. As GFR declines, excretion of oxalate and uric acid is impaired, with plasma oxalate rising from <2 µmol/L to 10–30 µmol/L in CKD G3-G4 [95]. Hyperoxaluria contributes to increased supersaturation of CaOx in the urine, which is a key factor in stone formation in the general population [96]. However, in CKD patients with hyperoxaluria, the relationship between oxalate levels and stone formation becomes more complex. The combination of reduced urinary calcium excretion, low citrate levels, and metabolic acidosis creates an environment that paradoxically reduces the likelihood of CaOx stone formation. Instead, hyperoxaluria in CKD patients contributes more prominently to kidney damage through oxalate-induced tubular injury, inflammation, and fibrosis rather than urolithiasis [16,37,66,67,68,88].

CKD-mineral and bone disorder (CKD-MBD) further complicates the picture. Elevated serum phosphate, vitamin D deficiency, and secondary hyperparathyroidism disrupt calcium–phosphate homeostasis, promoting ectopic calcification [97,98,99]. Treatments like phosphate binders, while addressing CKD-MBD, may inadvertently influence stone risk, complicating dietary and pharmacological stone prevention [100,101].

Together, the bidirectional relationship between nephrolithiasis and CKD emerges from shared risk factors (hypertension, diabetes, obesity, and metabolic syndrome) and diverse mechanisms, including obstruction, crystal-induced injury, infection, metabolic imbalances, and gut–kidney axis interactions, each driving kidney damage and altering stone risk profiles. These processes, amplified by gut microbial shifts and *SLC13A2* and *SLC26A1* transporter dysregulation, create a vicious cycle where stones accelerate CKD progression while declining GFR modifies lithogenesis, shifting stone composition and exacerbating tubular stress. This complexity challenges therapy to prevent stones while preserving kidney function, especially in patients with CKD.

## 3. Current Therapeutic Strategies: Benefits and Risks in CKD

### 3.1. Iatrogenic Interventions

Urological interventions, such as extracorporeal shock wave lithotripsy (ESWL), percutaneous nephrolithotomy (PCNL), ureteroscopy (URS), and ureteral stenting, are critical for managing nephrolithiasis, yet they carry iatrogenic risks that may contribute to kidney injury and influence CKD progression [102]. The evidence on their impact is mixed, with some studies reporting increased CKD risk [103,104] and others finding no significant long-term effect [105,106], highlighting the complexity of balancing stone removal with renal preservation.

In a recent single-center study, Candela et al. demonstrated that baseline CKD category, Charlson comorbidity index, operative time, and postoperative complications were associated with acute kidney disease following ESWL and URS [103]. Similarly, in a case-control study involving 204 patients with nephrolithiasis, the stone recurrence and number of surgical events were significantly associated with CKD [104]. Additionally, ESWL has been associated with decreased corticomedullary differentiation in 19.9% of patients and reduced parenchymal thickness in 11.6%, particularly after multiple sessions [107]. Mechanistically, ESWL generates shock waves (up to 2000–3000 pulses, 18–24 kV) that fragment stones but induce microvascular trauma, releasing ROS via NADPH oxidase, leading to tubular apoptosis and TGF-β/Smad3-mediated interstitial fibrosis detectable within weeks in experimental models [108,109].

PCNL remains the standard surgical approach for managing large or complex renal calculi [110,111]. Although most studies suggest that PCNL stabilizes or even improves kidney function in patients with CKD [110,112], the procedure presents a delicate balance between stone clearance and preserving kidney function. For instance, Izol et al. demonstrated an increase in GFR from 48.7 to 59.1 mL/min/1.73 m^2^ over 12 months in a cohort of 280 CKD patients, highlighting its potential benefits [111]. However, this therapeutic approach is not without risks. CKD patients undergoing PCNL face a higher likelihood of complications such as bleeding, infection, and AKI compared to those with normal renal function [110]. AKI post-PCNL occurs in approximately 24.9% of cases, with advanced age being a significant contributing factor [113]. Moreover, multi-access PCNL exacerbates the risk of renal deterioration due to increased surgical stress and parenchymal injury. For patients in advanced CKD (G4, G5), this dilemma becomes even more pronounced. Higher complication rates, prolonged recovery, and reduced stone clearance are frequently observed due to comorbid conditions, such as diabetes and hypertension, as well as intraoperative challenges, like impaired visualization from excessive bleeding [114,115].

Ureteroscopy, involving direct mucosal manipulation, causes mechanical abrasions and ischemia–reperfusion injury, elevating IL-1β and TNF-α via NF-κB activation and recruiting macrophages that amplify fibrosis [116]. Ureteral stents, used to relieve obstruction, serve as bacterial niduses, increasing UTI risk by two- to threefold [117,118]. Biofilm formation on stent surfaces facilitates bacterial adhesion, persistence, and immune evasion, contributing to recurrent or refractory infections [119].

However, the long-term impact of these interventions on CKD development remains controversial. A large retrospective cohort study of 1340 patients with urolithiasis found that urological procedures did not significantly increase the risk of CKD (HR 1.08 [0.77–1.49]) [105]. Interestingly, the same study reported an increased risk of elevated serum creatinine associated with urological procedures (HR = 1.49 [1.19–1.85]). This suggests that while these interventions may cause short-term changes in kidney function markers, they may not substantially increase the risk of CKD development in the long term. The American Urological Association (AUA) and European Association of Urology (EAU) guidelines recommend ESWL as a first-line option for small-to-medium renal stones (<20 mm) and URS for ureteral stones, while PCNL is the preferred first-line treatment for large or complex stones (>20 mm) in patients with normal kidney function; however, their use in CKD lacks specific endorsement due to heightened complication risks and limited long-term outcome data [120,121]. The mixed evidence regarding the long-term renal effects of urological interventions emphasizes the need for individualized risk assessment and careful procedural planning. Factors such as baseline kidney function, comorbidities, the procedure type, and its frequency should be considered when deciding on the most appropriate intervention strategy. Ongoing research into biomarkers and long-term outcomes may help develop more personalized approaches to stone management and renal preservation [116,122]. Thus, while iatrogenic interventions remain a crucial tool for stone management, careful patient selection and perioperative strategies are essential to balance effective stone removal with the preservation of kidney function.

### 3.2. Fluid Intake

Increasing fluid intake to achieve a urine output >2 L/day stands as a foundational strategy for kidney stone prevention, diluting urinary solutes and reducing recurrence by 50–60% (RR 0.45, 95% CI 0.24–0.85) in individuals with preserved kidney function [123,124]. A meta-analysis by Xu et al. has found that each 500 mL increase in water intake significantly reduces kidney stone risk (RR = 0.93, 95% CI: 0.87–0.98) [125]. It has been demonstrated that adequate hydration decreases urine acidity and prevents the supersaturation of minerals in urine, which is essential for avoiding nucleation and crystal growth [125,126]. Based on this and other well-proved data, guidelines recommend a daily fluid intake of 2.5–3 L to prevent kidney stone recurrence [121]. In CKD, however, where GFR is reduced, this approach becomes a conundrum [127]. Excess fluid risks volume overload, particularly in advanced CKD, exacerbating hypertension and tubular stress [128]. CKD also impairs urinary concentration, complicating whether increased volume reflects therapeutic benefit or disease progression [129]. Population-based studies have consistently shown that higher water intake is associated with a lower prevalence of CKD and slower GFR decline [128,130,131]. Increased hydration may help suppress plasma levels of vasopressin, which is linked to kidney damage, potentially slowing CKD progression [130]. However, a recent cohort study, including 1265 CKD patients with a mean GFR of 32 mL/min/1.73 m^2^, has found that both low (<0.5 L/day) and high (>2.0 L/day) plain water intake were associated with faster GFR decline and higher risk of kidney failure compared to moderate intake (1.0–1.5 L/day) [129]. These findings suggest hydration may slow CKD progression in early stages but pose risks in advanced stages due to fluid overload. Thus, water intake should be tailored, with recommendations of 1.5–2.0 L/day in moderate CKD and 1.0–1.5 L/day in advanced CKD, monitored for signs of edema and hypertension.

While water remains the primary fluid for stone prevention, citrus-based fluids like lemon juice (providing 50–100 mL/day citrate) [132] and caffeinated beverages like coffee or tea [133] may further reduce stone risk by increasing urinary citrate or altering urine chemistry, as supported by observational data in non-CKD populations. In CKD, however, their use requires caution due to the potassium content in citrus fluids (risking hyperkalemia in GFR < 30 mL/min/1.73 m^2^) and potential sodium or sugar loads in processed drinks, necessitating individualized assessment [134]. These alternatives highlight the need for tailored fluid strategies beyond water to optimize stone prevention while managing CKD progression.

### 3.3. Dietary Modifications

Dietary modifications are a cornerstone of kidney stone prevention, targeting specific lithogenic factors to reduce recurrence risk and offering potential benefits for kidney function [135,136]. Reducing sodium intake to <2 g/day decreases urinary calcium excretion by 20–40 mg/day, significantly lowering calcium stone risk [17,137]. Limiting dietary oxalate to <100 mg/day curbs hyperoxaluria, with studies showing a 20–40% reduction in urinary oxalate in adherent patients [138,139]. Increasing citrate through high fruit and vegetable intake (50–100 mg/day) inhibits stone formation by chelating calcium and raising urine pH, reducing recurrence by up to 25% [140,141]. Moderating animal protein to 0.8 g/kg/day reduces uric acid and calcium excretion, addressing both uric acid and calcium stone risks (RR 0.71, 95% CI 0.52–0.96) [135,138,142]. These strategies leverage dietary control over urinary supersaturation and crystal formation, key mechanisms outlined above.

In CKD patients, sodium restriction (<2 g/day) not only reduces urinary calcium for stone prevention but also lowers blood pressure and proteinuria, aligning with hypertension management guidelines [143]. However, careful monitoring is required to prevent hyponatremia, especially in patients with fluid overload or those on diuretics [144,145]. Hyponatremia prevalence varies widely and increases with CKD progression, affecting up to 10–30% of patients with G5 CKD [144,146]. Both baseline and time-dependent hyponatremia are associated with a higher risk of kidney failure in ambulatory CKD patients under 65 years (baseline HR 1.30, 95% CI 1.03–1.64; time-dependent HR 1.36, 95% CI 1.09–1.70) [145]. Sodium restriction thus offers greater benefits in early CKD, where it can complement fluid intake to manage stone risk and hypertension, but in advanced stages, it must be balanced with adequate hydration to prevent dehydration and hyponatremia, highlighting the need for stage-specific adjustments.

Although high oxalate intake is significantly associated with CKD progression [147,148], a low-oxalate diet may also pose some risks in CKD. Such diets often limit oxalate-rich plant-based foods, potentially reducing dietary fiber, essential vitamins, and antioxidants that benefit cardiovascular and kidney health [149,150]. In a recent study by Wu et al., a 31% lower risk of CKD has been shown in patients with hyperuricemia who adhere to a high plant-based diet [149]. Moreover, excessive calcium restriction in CKD patients following low-oxalate diets can paradoxically worsen hyperoxaluria by increasing intestinal oxalate absorption, contributing to secondary oxalate nephropathy and accelerating CKD progression [66].

Similarly, citrate-rich foods may present a potential risk in CKD due to their high potassium content, particularly in patients with GFR < 30 mL/min/1.73 m^2^. Potassium-rich diets are traditionally restricted in CKD patients to prevent hyperkalemia [134,151]. However, recent studies challenge this view, showing that controlled potassium-rich diets in pre-dialysis CKD patients lead to only a modest increase in serum potassium (0.2–0.4 mmol/L), suggesting that the risk may be overstated in stable patients [151,152]. Nevertheless, caution is necessary. Impaired potassium excretion, combined with medications such as angiotensin-converting enzyme inhibitors (ACEi), can still lead to life-threatening arrhythmias in high-risk individuals [151,152]. Therefore, careful monitoring and personalized dietary adjustments remain essential.

Beyond its role in stone prevention, protein restriction (0.6–0.8 g/kg/day) aligns with CKD guidelines and reduces uremic toxins, potentially slowing CKD progression [153]. However, in advanced CKD, excessive protein restriction risks protein-energy wasting, increasing malnutrition prevalence by 30–40% in this population [154]. This underscores the need for careful nutritional monitoring to balance the benefits of toxin reduction and stone prevention against the risk of malnutrition, particularly in later CKD.

A special concern in CKD is the management of phosphate balance, which plays a key role in both kidney stone formation and CKD-MBD progression. Restricting dietary phosphate (800–1000 mg/day) has been shown to lower serum phosphate levels by 0.5–1.0 mg/dL, reducing CKD-MBD risk and vascular calcifications [155]. However, limiting phosphate-rich foods like dairy reduces calcium intake, increasing oxalate absorption by 10–20% and raising calcium oxalate stone risk [156]. Furthermore, calcium-based phosphate binders may exacerbate hyperoxaluria by binding calcium [37]. Thus, balancing phosphate restriction with adequate calcium (800–1200 mg/day), alongside monitoring serum phosphate, calcium, and urinary oxalate, is crucial to prevent unintended stone formation and nutritional deficits in CKD.

### 3.4. Pharmacological Interventions

Pharmacological interventions play a pivotal role in kidney stone prevention by targeting urinary chemistry, but their use in CKD patients requires careful consideration of renal safety.

#### 3.4.1. Thiazides

Thiazide diuretics, such as hydrochlorothiazide (HCTZ, 25–50 mg/day) and chlorthalidone, are traditionally used to reduce urinary calcium excretion by 100–150 mg/day through enhanced proximal tubular reabsorption, aiming to prevent calcium stone recurrence [157]. However, the recent NOSTONE trial, a double-blind, placebo-controlled study, challenges this efficacy, showing no significant difference in stone recurrence across HCTZ doses of 12.5 mg, 25 mg, or 50 mg daily compared to placebo in patients with recurrent calcium stones [158]. Despite reduced urinary calcium, inconsistent effects on oxalate and citrate levels, alongside unchanged urine supersaturation ratios, may explain the lack of benefit. In contrast, recent studies have revitalized interest in thiazides for hypertension management in CKD, a key shared risk factor for nephrolithiasis and CKD progression. The Chlorthalidone in Chronic Kidney Disease (CLICK) trial, a double-blind, randomized, placebo-controlled study in 160 patients with G4 CKD (eGFR 15–29 mL/min/1.73 m^2^), demonstrated that chlorthalidone significantly reduced 24 h ambulatory blood pressure by 10.5/3.1 mmHg at 12 weeks compared to placebo, while also reducing albuminuria by 40–45%, suggesting a potential renoprotective effect [159]. A 2023 systematic review and meta-analysis further confirmed these findings, reporting a 15 mmHg reduction in mean arterial pressure in randomized trials of thiazides in advanced CKD (G3b-5), with observational studies showing a 10–15 mmHg systolic and 5–10 mmHg diastolic reduction [160]. Real-world evidence supports their tolerability, with a 2024 retrospective study finding that 83.8% of CKD G3b-G4 patients continued thiazides at 1 year and 71.6% at 2 years, with discontinuation rates (16.2% at 1 year, 28.4% at 2 years) lower than those for ACEi [161]. Additionally, a 2023 retrospective study in Taiwan involving 8501 CKD patients on thiazides reported improved cardiovascular and renal outcomes, though specific blood pressure data were not provided [162]. Despite these benefits, thiazides in CKD pose risks of volume depletion and electrolyte imbalances, with a 6.6–17% incidence of hypokalemia and hyponatremia in G3-G5 CKD, as well as a transient eGFR decline that reflects hemodynamic changes rather than true injury [159,160,163]. The NOSTONE trial also reported increased adverse events with HCTZ, including new-onset diabetes, gout, and elevated creatinine, highlighting safety concerns in CKD [158]. Per guidelines from the AUA and EAU, thiazides are a first-line therapy for calcium stone prevention in patients with hypercalciuria and normal kidney function, but their use in CKD is off-label and primarily supported for hypertension management rather than stone prevention [120,121]. Thus, while thiazides offer significant antihypertensive benefits in CKD, their role in stone prevention remains uncertain, necessitating careful monitoring and consideration of alternative strategies.

#### 3.4.2. Potassium Citrate

Potassium citrate (20–60 mEq/day) is a mainstay for preventing uric acid and calcium oxalate stones by alkalinizing urine (pH > 6.5) and increasing citrate excretion by 200–300 mg/day, reducing stone recurrence by 60–75% (RR 0.25, 95% CI 0.14–0.44) in patients with preserved kidney function [164]. In CKD, however, its use is complicated by several risks. Potassium supplementation raises plasma potassium levels by approximately 0.4 mmol/L, with hyperkalemia prevalence reaching 11% in G3b–G4 CKD patients [165]. Hyperkalemia risk in CKD is heightened due to reduced GFR and often concurrent use of RAAS inhibitors, which further impair potassium excretion. A 2017 population-based cohort study of 157,766 CKD patients in Denmark found that 31% of patients with G4 CKD and 42% with G5 CKD experienced hyperkalemia within the first year, with RAAS inhibitor use increasing the risk (prevalence ratio 1.45, 95% CI 1.42–1.48) [166]. Although this study did not specifically evaluate potassium citrate, the risk of hyperkalemia in advanced CKD suggests caution, as potassium citrate directly contributes to potassium load. A case report of two renal transplant patients highlighted this risk, noting acute hyperkalemia (serum potassium 7.67 and 6.05 mmol/L) after initiating potassium sodium hydrogen citrate at 10 g/day, requiring urgent interventions like hemofiltration [167].

An additional concern is over-alkalinization, as excessive citrate intake may raise urine pH above 7, promoting the formation of calcium phosphate stones, particularly in CKD patients with CKD-MBD, where alkaline urine favors calcium phosphate precipitation [168]. Krieger et al., using a hypercalciuric rat model, found that potassium citrate increased urine pH, oxalate, and phosphate levels, leading to higher calcium phosphate supersaturation despite lower urinary calcium, suggesting a complex effect that may not always reduce stone risk in calcium phosphate stone formers [168]. Clinical data in humans also indicate this risk, showing that potassium citrate’s pH-raising effect could negate its benefits in calcium phosphate stone formers by increasing calcium phosphate saturation, though it did not quantify the incidence of stone formation [169]. Furthermore, potassium citrate can exacerbate metabolic alkalosis in advanced CKD, where bicarbonate retention is impaired, potentially leading to complications like hypocalcemia and arrhythmias. To mitigate these risks, lower doses (e.g., 10–20 mEq/day) and alternative formulations, such as sodium citrate, have been explored, though sodium citrate may increase sodium load and exacerbate hypertension in CKD patients [170]. The AUA and EAU guidelines recommend potassium citrate as a first-line treatment for uric acid and calcium oxalate stones with hypocitraturia or acidic urine in patients with normal kidney function, but its use in CKD requires careful potassium monitoring and is not specifically endorsed for advanced stages [120,121]. Regular monitoring of serum potassium, urine pH, and calcium levels is essential, with adjustments based on GFR category and concurrent medications, to balance stone prevention with renal safety.

#### 3.4.3. Allopurinol

Allopurinol (100–300 mg/day), an xanthine oxidase inhibitor, reduces uric acid production, lowering uric acid stone risk by 50–60% (RR 0.42, 95% CI 0.29–0.61) in hyperuricosuric patients with preserved kidney function [17]. Beyond stone prevention, allopurinol may offer renoprotective benefits in CKD by mitigating oxidative stress. A recent meta-analysis found that allopurinol significantly slowed GFR decline compared to placebo, with a significant increase in GFR and reduction in serum uric acid levels [171]. However, its use in CKD carries notable risks. Allopurinol has been associated with AKI, with 5–10% of patients in CKD G3–G4 experiencing elevated creatinine levels [172,173]. Additionally, allopurinol hypersensitivity syndrome (AHS) is a rare but severe reaction occurring in 2–3% of patients, characterized by rash, eosinophilia, and multi-organ dysfunction, with a 20–25% mortality rate [174]. The risk is particularly high in patients with CKD, heart disease [175], and those carrying the HLA-B*5801 allele, especially in Asian populations, warranting genetic screening in high-risk groups [166]. To minimize toxicity, careful dose adjustment is essential in CKD, with guidelines recommending a maximum dose of 100 mg/day for patients with GFR < 30 mL/min/1.73 m^2^. The AUA and EAU guidelines position allopurinol as a second-line therapy for uric acid stone prevention, recommended only after dietary modification and urinary alkalinization fail, with no specific endorsement for CKD patients due to limited evidence [120,121]. Thus, while allopurinol provides dual benefits for stone prevention and potential renal protection, its risks in CKD necessitate individualized dosing and vigilant monitoring.

In summary, current therapeutic strategies for kidney stone prevention in CKD patients, including urological interventions, fluid intake, dietary modifications, and pharmacological treatments, offer both benefits and risks that require careful consideration. While these approaches can aid in kidney stone prevention, their impact varies based on the CKD stage and patient-specific factors. A personalized, evidence-based approach is essential to balance efficacy with renal preservation, minimizing complications while optimizing patient outcomes. Table 2 consolidates these strategies, detailing their stone prevention benefits, CKD progression impacts, and specific risks in CKD to guide tailored clinical decision-making.

## 4. Dual-Purpose Therapeutic Strategies

The limitations of current therapeutic strategies for nephrolithiasis in CKD highlight the need for innovative therapies that simultaneously prevent kidney stones and slow CKD progression. Dual-purpose strategies integrate emerging innovations with repurposed interventions to target shared pathophysiological mechanisms. Supported by preclinical and early clinical evidence, these approaches promise a precision medicine framework to optimize outcomes in CKD patients with nephrolithiasis.

### 4.1. Minimally Invasive Technologies

Advancements in minimally invasive technologies aim to improve stone clearance while minimizing kidney injury, thereby reducing the risks associated with traditional ESWL and PCNL [176]. Ultrasound-guided micro-PCNL uses smaller access sheaths (4.8–10 Fr vs. 24–30 Fr), which lowers the risk of bleeding and AKI in kidney stone patients compared to standard PCNL [177,178]. Surag et al. identified a tract size greater than 24 Fr as a significant predictor of severe bleeding requiring angioembolization after PCNL, whereas smaller tracts (<18 Fr) showed a 50% reduction in bleeding risk due to less parenchymal trauma [178]. A recent prospective study of 46 patients with CKD G3–G5 and nephrolithiasis reported a 90% stone-free rate, with significant improvement in GFR over six months postoperatively [179]. However, uncertainties persist regarding the generalizability of these findings, as the study’s small sample size and lack of a control group limit conclusions about long-term renal outcomes. Additionally, the technical complexity of micro-PCNL requires specialized equipment and expertise that may restrict its widespread adoption, particularly in resource-limited settings. In advanced CKD, comorbidities such as coagulopathy or impaired wound healing could still elevate complication rates, necessitating further investigation into patient selection criteria.

Laser-based stone disintegration, such as holmium laser lithotripsy with real-time perfusion monitoring, achieves a 95% stone-free rate with minimal kidney function impact (creatinine rise <0.1 mg/dL) in non-CKD cohorts, suggesting a potential to reduce fibrosis risks seen with URS [180,181]. The precise energy delivery minimizes thermal injury to surrounding tissues, a key advantage over traditional URS, which can induce inflammation and scarring [181]. Yet, CKD-specific trials are lacking, leaving uncertainty about its efficacy and safety in patients with reduced GFR, where baseline tubular vulnerability might amplify even minor insults.

### 4.2. Microbiota-Based Interventions

The gut microbiota’s role in nephrolithiasis and CKD progression has spurred interest in microbiota-based interventions that target dysbiosis. Microbiota modulation, through probiotics, prebiotics, synbiotics, and fecal microbiota transplantation (FMT), aims to restore eubiosis, reduce uremic toxins, and modulate oxalate metabolism, offering dual benefits for stone prevention and CKD management [182,183,184]. Experimental studies have demonstrated robust efficacy in reducing stone risk and slowing CKD progression, but clinical outcomes in humans are less consistent, underscoring challenges in translating preclinical success to patient care.

Probiotics, including Oxalobacter formigenes, Lactobacillus, and Bifidobacterium species, target gut oxalate degradation to reduce its absorption and urinary excretion while also mitigating CKD progression through anti-inflammatory and toxin-lowering effects [183,185]. Early rat studies showed that O. formigenes significantly reduced urinary oxalate and crystal deposition in hyperoxaluric models [186,187]. More recently, Verhulst et al. have confirmed that O. formigenes (strain HC-1) prevented ethylene glycol-induced increases in plasma oxalate and nephrocalcinosis in rats, reducing plasma oxalate by 30% and renal crystal deposition by 40% over 3 weeks [188]. Lactobacillus and Bifidobacterium species degrade oxalate and produce SCFAs, reducing inflammation and uremic toxins [185]. Darilmaz et al. have demonstrated that synbiotic supplementation (inulin plus Lactobacillus) increased oxalate degradation by 30% [189]. In CKD models, a probiotic containing Lactobacillus acidophilus and a synbiotic with inulin reduced urea, creatinine, C-reactive protein (CRP), and fibrosis in 5/6 nephrectomy rats, slowing disease progression [190]. Similarly, Lactobacillus johnsonii alleviated kidney injury and fibrosis in 5/6 nephrectomy and unilateral ureteral obstruction rats [191]. Synbiotics, combining probiotics and prebiotics like inulin, enhance oxalate degradation (up to 30%) and shift microbial metabolism, as shown by Darilmaz et al. [186].

Clinically, prebiotics and synbiotics promote SCFA production, improving gut barrier integrity and reducing serum levels of protein-bound uremic toxins, systemic inflammation, and oxidative stress [192,193]. A recent meta-analysis found that probiotics and synbiotics significantly lowered blood urea nitrogen, CRP, and inflammatory cytokines (TNF-α, IL-6, IL-18), suggesting a protective role against CKD-related inflammation [184]. However, human trials show less robust effects than preclinical models, with no significant improvements in GFR or serum creatinine, possibly due to variability in ethnicity, age, and GFR category [184,194]. Therefore, larger, controlled trials are needed to validate these interventions across CKD stages and stone types.

Fecal microbiota transplantation (FMT) offers a comprehensive microbial reset. In preclinical studies, FMT from healthy donors to germ-free mice reduced renal CaOx crystal deposition by 40% and urinary oxalate excretion by 24% [195]. In another study, FMT via colonoscopy (100 g donor stool) reduced urinary oxalate excretion by 20% in experimental rats [196]. Clinical studies are scarce and less pronounced. For example, oral FMT capsules from optimized donors (e.g., high Bifidobacterium adolescentis) achieved 67% microbiota engraftment in immunocompromised patients, demonstrating safety and potential for human application [197]. However, FMT’s complexity, including donor selection, delivery method (oral vs. colonic), and infection risks, limits scalability, and stone-specific human trials remain lacking.

### 4.3. Sodium–Glucose Cotransporter 2 Inhibitors

Sodium–glucose cotransporter 2 (SGLT2) inhibitors, including empagliflozin, dapagliflozin, and canagliflozin, are a well-established pharmacological class for renoprotection in CKD, with their benefits in slowing renal decline widely recognized [198,199]. Increasingly, evidence suggests these agents also reduce nephrolithiasis risk by modulating urinary chemistry, positioning them as a dual-aim therapy for CKD patients with kidney stones [200,201]. While their renoprotective mechanisms are well-documented, growing preclinical and clinical data highlight their potential to alter stone risk factors, though stone-specific outcomes remain exploratory and require further validation.

SGLT2 inhibitors influence urinary stone risk by inhibiting proximal tubular glucose reabsorption, which shifts sodium and bicarbonate handling to increase urinary citrate and pH, key inhibitors of stone formation [201,202,203]. Clinical evidence for stone risk reduction is accumulating, building on their known renoprotective foundation. The EMPA-REG OUTCOME study, involving over 15,000 type 2 diabetes patients, showed a 49% lower risk of nephrolithiasis among those receiving empagliflozin compared to placebo or other glucose-lowering therapies [204]. A meta-analysis of five clinical trials involving over 11 million patients found nephrolithiasis occurred in 1.27% of those taking SGLT2 inhibitors versus 1.56% in control groups, representing a 40% decreased risk compared with placebo and a 34% decreased risk compared with active comparators like GLP-1 receptor agonists or DPP-4 inhibitors [202]. A large-scale Japanese study involving 1,538,198 diabetic patients showed significantly lower nephrolithiasis prevalence among SGLT2 inhibitor prescribers compared to non-users [205]. These data align with mechanistic insights from a study by Harmacek et al., which demonstrated that patients on SGLT2 inhibitors exhibited significantly higher urinary citrate levels and a more alkaline urinary pH compared to controls [201]. These findings indicate that SGLT2 inhibitors could benefit CKD patients prone to CaOx or UA stones, particularly those with metabolic comorbidities. Beyond stone risk, they reduce albuminuria by 30–50% and CVD events, enhancing CKD management [198]. In non-diabetic CKD, the pilot SWEETSTONE trial (n = 50, G3) showed dapagliflozin increased urine volume by 300–500 mL/day and citrate in both CaOx and UA stone formers (60% and 40%, respectively), suggesting these benefits may extend beyond diabetic populations [206]. These findings indicate that SGLT2 inhibitors could benefit CKD patients prone to CaOx or UA stones, particularly those with cardiovascular comorbidities [198,207].

Despite these promising results, several limitations warrant caution. Reductions in stone recurrence (20–25%) and variability in urinary citrate and pH responses (15–30%) vary widely and may depend on factors like CKD category, baseline acidosis, or dietary habits, undermining claims of consistent efficacy across all patients [200]. In addition, safety concerns arise. For instance, the DAPA-CKD trial reported a 5–10% incidence of UTIs and volume depletion with dapagliflozin [198]. Zang et al. have found an increasing risk of contrast-associated AKI in diabetic patients with short-term SGLT2 inhibitor use [208]. Finally, although SGLT2 inhibitors are extensively studied in diabetic CKD, their efficacy and safety in non-diabetic, non-hypertensive CKD patients prone to kidney stones remain largely untested. Thus, while SGLT2 inhibitors present a compelling option for concurrently managing CKD and nephrolithiasis risk, their application should be tailored to individual patient profiles, weighing benefits against potential risks and costs.

### 4.4. Omega-3 Fatty Acids

Omega-3 fatty acids (e.g., eicosapentaenoic acid [EPA] and docosahexaenoic acid [DHA], 1–2 g/day), commonly obtained through habitual fish oil use or fatty fish consumption, offer a multifaceted approach by modulating urinary chemistry and renal inflammation [209]. Preclinical studies demonstrate a 15–20% reduction in urinary calcium and oxalate excretion, likely via altered membrane phospholipid composition and reduced activity of stone-promoting enzymes like phospholipase A2 [209,210]. Concurrently, omega-3s reduce TGF-β expression by 40–60% in the remnant kidney (5/6 nephrectomy) and unilateral ureteral obstruction models, limiting fibroblast activation and extracellular matrix production [211,212]. Broader evidence suggests omega-3 fatty acids may reduce inflammation and slow CKD progression, particularly through mechanisms like suppression of mesangial cell activation and modulation of inflammatory pathways [213,214]. Furthermore, regular fish oil use was associated with a lower risk of new-onset kidney stones in participants with low or intermediate genetic risk of kidney stones, suggesting a preventive role modulated by genetic predisposition [215]. These dual effects stem from omega-3s’ production of anti-inflammatory resolvins and protectins, which may counteract the oxidative stress implicated in both stone formation and CKD progression [216]. However, uncertainties remain: the optimal dose for CKD patients is unclear, with trials ranging from 1–4 g/day showing variable efficacy, and the pilot’s small size and short duration limit conclusions about long-term stone recurrence or GFR benefits [209,217]. The genetic risk finding [215] requires validation in CKD cohorts, where polygenic stone risk profiles may differ, and the magnitude of risk reduction remains unspecified. In advanced CKD, impaired lipid metabolism could reduce omega-3 bioavailability, and potential interactions with anticoagulants warrant caution, especially in patients with comorbidities like diabetes or hypertension [217]. Larger, stage-specific RCTs are needed to confirm these preliminary findings and establish dietary guidelines.

### 4.5. Magnesium

Magnesium supplementation, though not a cornerstone of current AUA/EAU guidelines for stone prevention, emerges as a promising dual-purpose strategy by leveraging its ability to modulate CaOx stone formation and CKD-related mineral dysregulation. Magnesium inhibits CaOx crystallization by forming soluble oxalate complexes, reducing urinary supersaturation, an effect amplified in acidic environments with citrate co-administration [218,219]. It also curbs dietary oxalate absorption in the gut, enhanced by citrate-rich foods [218]. In CKD, magnesium regulates mineral metabolism by suppressing parathyroid hormone secretion, activating the calcium-sensing receptor, and limiting phosphate uptake, countering CKD-MBD [220]. Moreover, magnesium regulates pathways associated with inflammation, oxidative stress, and fibrosis, which are key factors in CKD pathogenesis [220,221]. Clinical evidence supports magnesium’s dual potential. Both magnesium oxide and magnesium citrate have been shown to significantly reduce urinary oxalate excretion in stone-forming patients [222]. Moreover, higher dietary magnesium intake (≥350 mg/day) is associated with a reduced risk of kidney stone formation, as demonstrated in a cross-sectional NHANES 2011–2018 analysis involving 19,271 U.S. adults [223]. In CKD G3–G4, daily supplementation with magnesium carbonate (360 mg/day) over 15 months improved bone mineral density and reduced vascular stiffness as measured by pulse wave velocity [224]. Dietary magnesium intake has also been linked to a lower risk of cardiovascular events (e.g., myocardial infarction and stroke) [225] and improved albuminuria [226]. These findings suggest magnesium may be particularly beneficial for CaOx stone formers with concurrent CKD-MBD. However, its clinical use is limited by the lack of large, randomized controlled trials. Current guideline omissions reflect limited and variable evidence, with urinary oxalate reductions ranging from 10–20% depending on dose (300–600 mg/day), formulation (oxide vs. citrate), and dietary factors. In advanced CKD (GFR < 30 mL/min/1.73 m^2^), magnesium accumulation poses a risk for hypermagnesemia, necessitating careful monitoring. Additional concerns include gastrointestinal side effects and potential interactions with anticoagulants, particularly in patients with diabetes or other comorbidities [220].

### 4.6. Noncalcium Phosphate Binders

Noncalcium phosphate binders, such as lanthanum carbonate and sevelamer, are established for managing hyperphosphatemia in CKD-MBD but show promise as a dual-purpose strategy. By binding dietary oxalate and phosphate in the gut, they reduce urinary oxalate and serum phosphate, potentially mitigating CKD progression. Preclinical studies have reported a 25% reduction in urinary oxalate and a 20% decrease in tubular crystal deposition in hyperoxaluric rats [227]. Despite their theoretical benefit in nephrolithiasis prevention, clinical studies on non-calcium phosphate binders for this purpose are scarce, likely due to their high cost. However, two recent case reports have demonstrated the efficacy of lanthanum carbonate in reducing both circulating and urinary oxalate levels while preventing recurrent nephrolithiasis in CKD patients [228,229]. Comprehensive clinical trials are needed to quantify their role in kidney stone prevention, evaluate long-term CKD outcomes, and refine their therapeutic applications beyond phosphate management.

## 5. Integrated Management and Future Directions for Nephrolithiasis in CKD

To address this therapeutic dilemma, a decision-making framework can be applied that integrates established approaches into a logical summary based on three key principles: (1) risk stratification, which assesses GFR, comorbidities, and stone type to identify high-risk patients; (2) stage-specific adjustments, tailoring therapy to kidney function and metabolic changes; and (3) monitoring, tracking urine chemistry, electrolytes, and GFR to adapt interventions. In addition, the integration of screening strategies, such as routine imaging and metabolic assessments, facilitates early detection of stone recurrence and CKD progression, reinforcing the importance of secondary prevention in this population. Table 3 provides a decision-making algorithm to navigate this balance, from aggressive prevention in early CKD to safety-focused care in advanced stages, incorporating dual-purpose options and monitoring triggers.

Despite significant advancements, several key challenges persist in managing nephrolithiasis in CKD. The long-term renal impact of urological interventions remains uncertain, with inconsistent findings regarding their effect on CKD progression [105,230]. The use of thiazides for stone prevention in CKD has been questioned in studies such as NOSTONE [158], and the safety of potassium citrate and allopurinol in advanced CKD remains a topic of ongoing investigation [170,171,173]. There is also a lack of consensus on optimal fluid intake and dietary thresholds across CKD stages, and the role of the gut–kidney axis in influencing both stone formation and kidney damage is still not well understood [36,86,124,127].

Therefore, future research should prioritize several key areas. First, longitudinal studies are essential to evaluate the long-term renal outcomes of repeated urological procedures and pharmacological interventions in CKD populations. This foundational research will provide critical insights into the safety and efficacy of current treatments over time. Following that, randomized controlled trials should refine fluid intake and dietary recommendations, particularly for patients in G3–G5 of CKD, while also exploring novel therapies that target oxalate metabolism and the gut microbiota. These trials will be instrumental in optimizing treatment strategies for nephrolithiasis in the context of CKD. Another critical area for investigation is biomarker discovery, which will help identify reliable indicators for predicting both stone recurrence and CKD progression. This advancement will enhance risk stratification and guide personalized treatment approaches. Finally, research into the gut–kidney axis is needed to better understand the role of gut microbiota in both stone formation and kidney function. Investigating interventions like probiotics or prebiotics could offer promising, holistic treatment strategies for patients.

Addressing these research gaps will strengthen evidence-based clinical guidance, enabling clinicians to more effectively balance stone prevention with kidney function preservation. As the global burden of nephrolithiasis and CKD continues to rise, integrating these strategies into clinical practice will help improve patient outcomes and advance multidisciplinary care for this complex patient population.

## 6. Conclusions

Nephrolithiasis associated with CKD presents a unique and increasingly relevant clinical issue that extends beyond traditional stone prevention paradigms. The interplay between lithogenic mechanisms and renal vulnerability necessitates a nuanced approach that balances the risk of stone recurrence with the imperative to preserve kidney function over the long term. As the systemic causes and renal consequences of kidney stones become increasingly apparent, it is clear that fragmented, specialty-specific models of care are insufficient to manage this dual burden.

An integrated, stage-specific framework is essential, allowing interventions such as hydration, dietary modification, and pharmacologic therapy to be appropriately tailored to kidney function and comorbidities. Emerging therapies, including SGLT2 inhibitors, microbiota-targeted strategies, and novel minimally invasive technologies, offer promise as dual-purpose solutions but require further clinical validation, particularly in advanced CKD cohorts.

To advance clinical practice, future research should prioritize high-quality randomized controlled trials and prospective cohort studies that assess both stone recurrence and CKD progression as primary outcomes. Additionally, interdisciplinary care models that bridge urology and nephrology must be strengthened to support individualized, risk-adapted management.

Ultimately, improving outcomes for patients with nephrolithiasis and CKD will require a paradigm shift—from siloed, disease-specific care toward a patient-centered approach that integrates metabolic, urologic, and nephrologic perspectives to ensure both efficacy and renal safety.

## Figures and Tables

**Table 1:** CKD stage-associated risk of nephrolithiasis and main drivers.

GFR Category	Dominant Stone Types	Biochemical Changes	Key Drivers
G1–G2	Calcium oxalate (69.3%) Calcium phosphate (11.9–19.7%)	Normal to slightly acidic urine (pH~6) Elevated urinary oxalate Early decrease in urinary calcium	Hyperoxaluria Declining GFR Normal to mild citrate levels
G3–G5	Uric acid (28.7%) Calcium oxalate (decreased)	Acidic urine (pH < 5.5) Reduced citrate Low urinary calcium Decreased magnesium and phosphorus	Metabolic acidosis Impaired oxalate/uric acid excretion CKD-MBD (high phosphate, secondary hyperparathyroidism)

Abbreviations: CKD-MBD: CKD-mineral and bone disorder; GFR: glomerular filtration rate.

**Table 2:** Summary of risks and benefits of current therapeutic strategies for nephrolithiasis in CKD.

Strategy	AUA/EAU Guideline Recommendations	Stone Prevention Benefit	CKD Progression Impact	CKD-Specific Risks/Considerations
**Urological Interventions**				
ESWL	1st-line for small-to-medium renal stones (<20 mm) in normal KF; no CKD-specific guidance	Fragments stones, reduces obstruction	Mixed evidence; may cause short-term kidney function changes	↓ corticomedullary differentiation, ↓ parenchymal thickness; AKI risk tied to GFR category
PCNL	1st-line for large/complex stones (>20 mm) in normal KF; lacks CKD endorsement	Clears large stones; stabilizes/improves GFR in CKD	Can improve GFR in CKD patients over time	AKI risk; ↑ bleeding/infection in GFR category G4–G5
URS	1st-line for ureteral stones in normal KF; limited CKD data	Relieves ureteral obstruction	AKI may occur, influenced by GFR category and comorbidities	Mucosal injury, fibrosis; infection risk
Ureteral Stenting	Adjunct to stone management; no CKD-specific guidance	Supports healing post-obstruction	Minimal long-term impact on CKD development	2–3x UTI risk; biofilm-related infections
**Fluid Intake**				
Water	2.5–3 L/d 1st-line for stone prevention in normal KF; CKD adjustment needed	Reduces recurrence by 50–60%; each 500 mL increase lowers risk	Slows GFR decline in early CKD; may worsen in advanced CKD with excess	Volume overload in G3–G5; tailored to 1.5–2 L/d (G3), 1–1.5 L/d (G4–G5)
Citrus-Based Fluids	Supports stone prevention via citrate in normal KF; no CKD-specific guidance	↑ Urinary citrate (50–100 mL/d); may ↓ recurrence	Limited direct impact; potassium may complicate advanced CKD	Hyperkalemia risk (GFR < 30)
Caffeinated Beverages	May reduce stone risk in normal KF; no CKD-specific guidance	May ↓ stone risk via urine dilution/altered chemistry	Minimal direct effect; unclear in CKD	Caffeine/sugar load; requires monitoring in CKD
**Dietary Modifications**				
Sodium (<2 g/d)	1st-line for stone prevention; aligns with CKD HTN management	↓ Urinary Ca^2+^ 20–40 mg/d; ↓ Ca stone risk	Reduces BP and proteinuria, aiding early CKD	Hyponatremia (10–30% in G5);
Oxalate (<100 mg/d)	1st-line for CaOx stones; CKD risks noted	↓ Urinary oxalate 20–40%	High oxalate worsens CKD; plant-based diets may mitigate	Limits fiber/antioxidants; ↑ oxalate absorption if Ca^2+^ low
Citrate (50–100 mg/d)	1st-line for stone inhibition; CKD K^+^ caution	↓ Recurrence by 25%	Limited direct effect; supports kidney health indirectly	Hyperkalemia risk (GFR < 30); K^+^ ↑ 0.2–0.4 mmol/L
Protein (0.8 g/kg/d)	1st-line for UA/Ca stones; CKD protein restriction supported	↓ UA/Ca^2+^	Slows progression by reducing uremic toxins	PEW (30–40% in advanced CKD)
Phosphate (800–1000 mg/d)	General stone prevention; KDIGO tailors for CKD-MBD	Indirect via CKD-MBD reduction	Reduces CKD-MBD progression	↑ Oxalate absorption 10–20% if Ca^2+^ low
**Pharmacological Interventions**				
Thiazides (25–50 mg/d)	1st-line for hypercalciuria in normal KF; off-label in CKD for BP	↓ Urinary Ca^2+^ 100–150 mg/d; NOSTONE: no recurrence benefit	Improves BP and albuminuria, potentially renoprotective	Hypokalemia/ hyponatremia (6.6–17%); transient eGFR decline
Potassium Citrate (20–60 mEq/d)	1st-line for UA/CaOx stones; CKD monitoring required	60–75% ↓ recurrence; ↑ citrate 200–300 mg/d	Hyperkalemia risk may complicate outcomes	Hyperkalemia (11% in G3b-G4); over-alkalinization (pH > 7)
Allopurinol (100–300 mg/d)	2nd-line for UA stones after diet/alkalinization; no CKD endorsement	50–60% ↓ UA stone risk	Slows GFR decline, offers renoprotection	AKI (5–10% in G3–G4); AHS (2–3%, 20–25% mortality)

Abbreviations: AHS: allopurinol hypersensitivity syndrome; AKI: acute kidney injury; AUA: American Urological Association; BP: blood pressure; Ca: calcium; CaOx: calcium oxalate; CKD: chronic kidney disease; CKD-MBD: CKD-mineral and bone disorder; d: day; EAU: European Association of Urology; ESWL: extracorporeal shock wave lithotripsy; GFR: glomerular filtration rate; HTN: hypertension; K^+^: potassium; KDIGO: Kidney Disease: Improving Global Outcomes; KF: kidney failure; PCNL: percutaneous nephrolithotomy; PEW: protein-energy wasting; PO_4_^3−^: phosphate; UA: uric acid; URS: ureteroscopy; UTI: urinary tract infection; ↓: decrease, ↑: increase.

**Table 3:** Decision-making algorithm for balancing stone prevention and CKD progression.

Step	GFR Category Consideration	Stone Prevention Priority	CKD Preservation Priority	Recommended Action
1. Assess Stone Risk	G1–G2: High recurrence risk	Reduce supersaturation	Monitor GFR decline	Fluids (2.5–3.0 L/day); CaOx: thiazides (25–50 mg/day), probiotics; UA: allopurinol (100–300 mg/day), K^+^ citrate (20–60 mEq/day); Struvite: antibiotics, ESWL
	G4–G5: Lower CaOx risk	Target specific stone types	Avoid overload	Fluids (1–1.5 L/day); CaOx: probiotics, magnesium (150–300 mg/day); UA: NaHCO_3_ (650 mg BID), allopurinol (50–100 mg/day); Struvite: antibiotics, micro-PCNL if obstructing
2. Evaluate CKD Risk	G3: Moderate progression	Balance efficacy vs. safety	Control BP, limit K^+^	Fluids (1.5–2 L/day); CaOx: thiazides (12.5–25 mg/day), probiotics; UA: allopurinol (100 mg/day), K^+^ citrate (10–20 mEq/day); Struvite: antibiotics, URS
	G4–G5: High progression	Minimize harm	Prioritize GFR stability	Avoid K^+^ citrate; CaOx: probiotics, noncalcium binders; UA: NaHCO_3_ (650 mg BID); Struvite: antibiotics, micro-PCNL if obstructing
3. Integrate Dual Therapy	All stages: Comorbidities present	Target shared mechanisms	Slow CKD progression	SGLT2i: 10 mg/day (G1–G4); Omega-3: 1–2 g/day; Probiotics: all stages; Magnesium: >350 mg/day (G1–G3), 150–300 mg/day (G4–G5); Noncalcium binders: for CKD-MBD, CaOx
4. Monitor and Adjust	All stages: Dynamic adjustment	Track recurrence (imaging 6–12 mo)	Assess trends (GFR 3–6 mo)	Adjust if K^+^ > 5.2 mmol/L (G3–G5), Mg^2+^ > 1.2 mmol/L (G4–G5); albuminuria rises, or edema; reassess 1–3 mo if off-target

Abbreviations: BID: twice daily; CaOx: calcium oxalate; ESWL: extracorporeal shock wave lithotripsy; K^+^: potassium; Mg²^+^: magnesium; SGLT2i: sodium–glucose cotransporter 2 inhibitors; PCNL: percutaneous nephrolithotomy; UA: uric acid; URS: ureteroscopy.

## Supplementary Materials

The following supporting information can be downloaded at: https://www.mdpi.com/article/10.3390/jcm14113678/s1, Supplementary File S1: Methods.
