# Supplementary material for: Balancing Stone Prevention and Kidney Function: A Therapeutic Dilemma
Source: J Clin Med. 2025 May 23;14(11):3678. doi: 10.3390/jcm14113678 (PMC12156816; doi:10.3390/jcm14113678)
Supplement: Supplementary file 1 [file jcm-14-03678-s001.zip › jcm-3630328-supplementary.pdf]

Supplementary File S1: Methods

Search Strategy and Databases

The literature search focused on studies published over the past decade (January 2015 to April 2025) to ensure the inclusion of relevant and up-to-date research. It is also supplemented by seminal pre-2015 studies cited in newer literature or critical to understanding nephrolithiasis and chronic kidney disease (CKD). PubMed, Scopus, and Web of Science databases were used for the search, selected for their comprehensive coverage of peer-reviewed biomedical literature. In addition, Google Scholar was used to capture recent publications that may not yet be listed in other databases.

Keywords

The following set of keywords was utilized to capture studies on nephrolithiasis, CKD, their epidemiology, mechanisms, screening, stone composition, urine biochemistry, and management strategies:

- **Condition-related terms**: “nephrolithiasis,” “urolithiasis,” “kidney stones,” “renal calculi,” “urinary stones,” “chronic kidney disease,” “CKD,” “renal impairment,” “kidney failure,” “CKD-MBD,” “arterial hypertension,” “diabetes,” “obesity,” “metabolic syndrome,” “end-stage kidney disease,” “ESKD.”
- **Epidemiological terms**: “prevalence,” “incidence,” “epidemiology,” “disease burden,” “morbidity.”
- **Mechanistic terms**: “pathophysiology,” “mechanisms,” “pathway,” “gut-kidney axis,” “metabolic acidosis,” “crystal-induced injury,” “oxidative stress,” “inflammation,” “fibrosis,” “oxalate,” “oxalic acid,” “uric acid,” “uricemia,” “acute kidney injury,” “AKI,” “obstruction,” “renal recovery,” “inflection.”
- **Stone composition terms**: “calcium oxalate,” “calcium phosphate,” “uric acid stones,” “struvite,” “cystine,” “brushite,” “stone type,” “mineral composition.”
- **Urine biochemistry terms**: “urinary pH,” “citrate,” “calcium,” “phosphate,” “magnesium,” “supersaturation,” “crystalluria,” “urine volume,” “urinary oxalate,” “urinary uric acid,” “urinary sodium,” “urinary potassium.”
- **Screening terms**: “screening,” “early detection,” “secondary prevention,” “urine chemistry,” “imaging,” “ultrasound,” “computed tomography,” “CT scan.”
- **Therapeutic terms**: “prevention,” “treatment,” “management,” “intervention,” “dietary modification,” “pharmacological therapy,” “fluid intake,” “thiazides,” “potassium citrate,” “SGLT2 inhibitors,” “probiotics,” “symbiotics,” “gut microbiota transplantation,” “urological intervention,” “lithotripsy,” “ureteroscopy,” “percutaneous nephrolithotomy,” “PCNL,” “alkalinization therapy,” “allopurinol,” “febuxostat,” “citrate supplementation,” “low-oxalate diet,” “low-purine diet.”
- **Outcome-related terms**: “renal function,” “kidney function,” “GFR,” “GFR category,” “stone recurrence,” CKD stage,” “CKD progression,” “renal outcomes,” “mortality.”

Example search string: ("nephrolithiasis" OR "urolithiasis" OR "kidney stones" OR "renal calculi") AND ("chronic kidney disease" OR "CKD" OR "renal impairment" OR "ESRD") AND ("prevention" OR "treatment" OR "pathophysiology" OR "gut-kidney axis" OR "screening" OR "acute kidney injury" OR "epidemiology" OR "calcium oxalate" OR "urinary pH" OR "lithotripsy").

Inclusion/Exclusion Criteria

Studies were included based on the following criteria:

- **Publication Type**: Peer-reviewed articles, including cohort studies, randomized controlled trials, meta-analyses, systematic reviews, and narrative reviews.
- **Language**: English-language studies to ensure accessibility and consistency in interpretation.
- **Relevance**: Studies addressing the pathophysiology (e.g., shared risk factors, mechanisms of kidney damage), therapeutic strategies (e.g., fluid intake, dietary modifications, pharmacological interventions), or clinical outcomes (e.g., stone recurrence, CKD progression).
- **Study Population**: Clinical studies involving adults with nephrolithiasis, CKD, or both, across all GFR categories; experimental studies (animal or in vitro) included for mechanistic insights into processes like stone formation, tubular injury, or gut-kidney interactions.

Non-English language studies, articles on irrelevant topics, or with low-quality evidence (case reports, editorials), and studies from before 2015 were excluded unless they were foundational works that were cited in the included studies or were relevant to the historical context.

Data Synthesis

Data were synthesized narratively due to the review’s integrative scope, which combines pathophysiological mechanisms, clinical evidence, and therapeutic strategies. The synthesis prioritized studies offering actionable insights for clinicians and identifying research gaps.
